# The possible protective role of HLA B27 and relevant immune markers in Juvenile Idiopathic Arthritis patients

**DOI:** 10.12669/pjms.40.5.7915

**Published:** 2024

**Authors:** Farhana Shahzad, Romeeza Tahir, Faheem Shahzad, Nadeem Afzal

**Affiliations:** 1 Farhana Shahzad, MBBS, M.Phil. Immunology Assistant Professor of Immunology University of Child Health Sciences, The Children’s Hospital, Lahore, Pakistan; 2 Romeeza Tahir, MSC, M.Phil, PhD Immunology Assistant Professor of Immunology, University of Health Sciences, Lahore, Pakistan; 3 Faheem Shahzad, Senior Lab Manager, University of Health Sciences, Lahore, Pakistan; 4 Nadeem Afzal Professor of Immunology, Akhtar Saeed Medical College, Lahore, Pakistan

**Keywords:** JIA, HLA B27, Cytokines, B cells

## Abstract

**Background & Objectives:**

JIA is a disease with different immunological characteristics and a complicated genetic foundation. HLA B27 is a risk factor for the development of JIA, and its impact on immunopathogenesis of the disease is also an area of interest. To determine whether HLA B27 and immune markers varied between JIA patients and healthy population.

**Methods:**

This comparative cross-sectional study was conducted at Immunology Department of University of Health sciences (UHS), Lahore from February 2018 till August 2021. A total of (71) JIA patients and (34) healthy controls were enrolled. B cells were enumerated by flowcytometry, ELISA was used for serum cytokines estimation and HLA B27 allele was detected by SPSS polymerase chain reaction.

**Results:**

The HLA B27 allele was significantly more in the control group than in the patient group, suggesting it is a protective allele to prevent JIA. Peripheral blood B cell counts and percentages were significantly lower in the HLA B27 positive group than in the HLA B27 negative group of control population. Serum cytokine levels were not significantly different between the HLA B27 positive and HLA B27 negative allele of the two study populations.

**Conclusion:**

In this study B cells are different between the two groups of control population however; serum cytokines are comparable between the study groups. Though, it was indicated that HLA B27 may be a preventive allele in the onset of JIA.

## INTRODUCTION

JIA is a diverse group of arthritis divided into seven categories by The International League of Associations for Rheumatology (ILAR) characterized by swelling, heat, and pain, affecting children under the age of sixteen.^1^ It is more common in people of European heritage and less common in people of Asian descent due to geographical and population variations.^1^ The profile of JIA in the Indo-Pak subcontinent differs from that in the West, with polyarticular and oligoarticular JIA being the most prevalent in local population.^2^

JIA is thought to be a complex autoimmune illness affecting those with a genetic predisposition.^3^ HLA B27 is a complex allele that is associated with JIA.^4^ It has an immunological and inflammatory role in arthropathies, such as presentation of arthritogenic peptides, aberrant folding of surface heavy chains, misfolding, and enhanced intracellular microbial survival.^5^

The pathogenesis of JIA may be influenced by an imbalance of pro- and anti-inflammatory cytokines, with TNF-α, IL-1, and IL-6 being the most important modulators. These cytokines have critical immunological responses related to autoimmunity, persistent inflammation, tissue and cartilage degeneration, and narrowing and erosion of joint spaces.^6^ B cells may play a role in JIA pathogenesis, as evidenced by early onset of disease with B cell signatures in peripheral blood and frequent ANA positivity in JIA patients.^7^

Pediatric rheumatologists are likewise curious about this connection in the hope of enhancing better understanding of the disease. Therefore, the present study is designed to determine the HLA B27 allele, proinflammatory cytokines, and B cells in JIA patients.

## METHODS

This comparative cross-sectional study was conducted from February 2018 till August 2021. The sample size was calculated using data from De Jager et al., (2007), keeping the power of study equal to 90% and the level of significance equal to 5%.^8^ The study included 71 JIA patients from the Rheumatology Outpatient Department, The University of Child Health Sciences and Children’s Hospital, Lahore, Pakistan and 34 healthy children with no documented history of JIA or clinical symptoms. The diagnostic criteria for the patients were that they had arthritis of one or more than one joint, for more than six weeks, under the age of 16, and excluding other types of arthritis. The trial participants had no primary orthopedic issues, chronic illness, or taking no immune-modulating drugs.

### Ethical Approval:

The study was approved by the Ethical Committee and Research Board of the University with letter No. UHS / REG -17 / ERC / 2490. A written consent and a questionnaire from the parents or legal guardians of each participant were obtained and were informed about the details of the study.

From each participant 5-7 ml of blood was obtained in a 10ml disposable syringe under aseptic circumstances and each sample was separated into three parts. The first portion was transferred to an EDTA vacutainer tube (BD medical, USA) for flowcytometry, the second half was transferred to an EDTA vacutainer tube for DNA extraction, and the third section was placed in a yellow top gel vacutainer tube. Fresh serum was obtained by centrifuging samples at 3000 rpm for five minutes and stored at -80^o^C for cytokine measurement.

**Fig.1:**
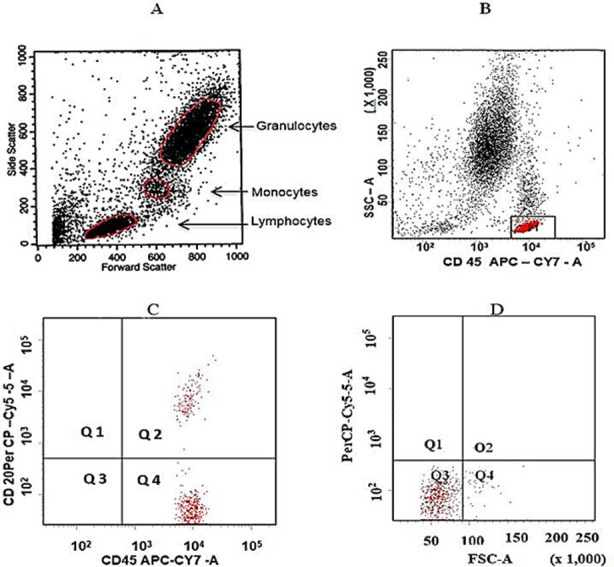
Identification and enumeration of CD20 B lymphocytes in peripheral blood. A- Based on the forward scatter and side scatter three populations of blood cells were identified (Granulocytes, Monocytes, and Lymphocytes). B- B cells were gated and identified by the expression of CD45on the cell surface of lymphocyte C- B cells were identified by surface expression of CD20 on the CD45 gated population (upper right quadrant D- Representative Isotype control dot plot

The Lyse-wash sample preparation procedure was used with whole blood. CD45 (APC CY7) and CD20 (PerCP) monoclonal antibodies (BD Biosciences USA), were used. Cells were analyzed with a FACS Canto II 6-color analyzer (BD Biosciences, California USA). Forward and side scatter was used to gate the lymphocyte population which was analyzed for B cell population. B cells were determined by using the gating strategy (Fig.1).

**Fig.2:**
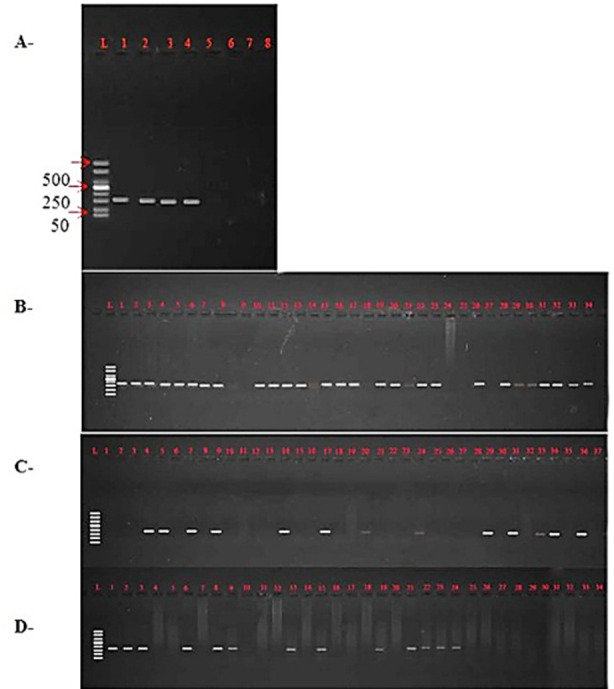
A- Optimization of HLA-B27 allele (149bp). L is a 50 bp DNA ladder (Thermo Scientific) Lane 1 to 8 contains known positive samples of HLA-B27 amplified at the following temperatures. 1= 56^0^C 2= 57^0^C 3 = 58^0^C 4= 59^0^C 5= 60^0^C 6= 61^0^C 7= 62^0^C 8= 63^0^C. Lane 1, 2, 3 and 4 showing positive bands of HLA-B27 allele. B- Amplification of HLA-B27 allele in the control group. L is a 50 bp DNA ladder (Thermo Scientific) Lane 1 to 34 showing results of 34 samples of the control group amplified for the HLA-B27 allele. C- Amplification of HLA-B27 allele in the patient Group. L is a 50 bp DNA ladder (Thermo Scientific) Lane 1 to 37 showing results of 37 samples amplified for the HLA-B27 allele. D- Amplification of HLA-B27 allele in the patient Group. L is a 50 bp DNA ladder (Thermo Scientific) Lane 1 to 34 showing results of 34 (38-71) samples amplified for HLA-B27 allele.

Serum Cytokines (Inteleukin-1, Inteleukin-6, TNF-α) were determined by commercially available quantitative sandwich enzyme immuno-assay kits (BT LAB, Birmingham, England). Genomic DNA was extracted from whole blood sample using phenol chloroform method. Amplification of HLA B27 allele was done by SSP-PCR. The quantity of extracted DNA was checked using a nano Drop 2000 spectrophotometer (Thermo Fisher Scientific Inc., USA). The amplified PCR products were electrophoresed on 2% agarose gel, stained with ethidium bromide, and viewed with a Bio-Rad Gel Doc XR+ Gel Documentation System (Bio-Rad Laboratories, Inc., Hercules, California, USA) (Fig.2). Sequence analysis of HLA B27 was done by Sanger sequencing from Lab genetix (Gene Insight Expert, Lahore, Pakistan) and results were checked by using program chromas version 2.6.6.

### Statistical Analysis:

SPSS version 26 for Windows was used to analyze study data. Median and interquartile range was calculated for non-normally distributed quantitative variables; Mann-Whitney rank sum test was used for comparison of variables between the two groups. Pearson Chi Square test was used to observe the association of alleles with disease. For categorical variables (such as allele) number and percentages were calculated. All statistical tests were two sided and a p-value of equal or less than 0.05 was considered statistically significant.

## RESULTS

The baseline clinical and lab characteristics of the study participants are summarized in Table-I.

**Table-I:** The baseline clinical and Lab characteristics of the study participants.

Variables		JIA patient group (n=71)	Healthy Controls (n=34)	p - Value^1^
Age	Median (IQR)^a^	11.00 (8.25-13.00)	10.50	0.95^b^
		(8.00- 13.25)
Gender n (%)^d^	Male	34(47.9%)	20 (58.8%)	0.30^c^
	Female	37 (52.1%)	14 (41.2%)
Duration of illness Median (IQR)		4.00 (2.00-6.00)	^e^NA	NA
Type of JIA	Poly	50 (70.4%) RF+ve=24 RF-ve=26	NA	NA
n (%)	Oligo	10 (14.0%) Persistant = 8 Extended = 2		
	Systemic	11(15.4%)		
Fever n (%)	Yes	34 (47.8%)	NA	NA
Morning Stiffness	Yes	46 (64.7%)	NA	NA
Deformatis	Yes	39 (54.9%)	NA	NA
Joint Pain	Yes	46 (64.7%)	NA	NA
Hepatomegaly	Yes	2 (2.8%)	NA	NA
Splenomegaly	Yes	5 (7.0%)	NA	NA
Rash	Yes	6 (8.4%)	NA	NA
Uveitis	Yes	2 (2.8%)	NA	NA
RAF (mg /dl) Median(IQR)		11.59 (7.30-33.48)	7.35 (5.88 - 8.90)	<0.001
CRP (mg /dl) Median(IQR)		6.80 (0.89-34.66)	0.38 (0.19 - 1.45)	<0.001
ESR (mm/hr)		40.00 (18.00-51.00)	8.00 (5.00 - 17.25)	<0.001
ANA positivity n (%)		26 (53.0%)	1(2.9%)	<0.001

a Inter-quartile range,

b p-value was determined by Mann-Whitney U-test,

c *p*-value was determined by Pearson’s chi-square test,

d number and percentage (frequency),

e not applicable,

1 p≤ 0.05 is considered statistically significant.

Frequency of HLA B27 in JIA patient and control groups and their association with JIA is shown in Table-II. HLA B27 was more prevalent in control group 30 (88.2%) than JIA patients group 27 (38.0%), with an odds ratio and confidence interval of 0.082 (0.026 - 0.258), significantly suggesting it as a protective allele to develop JIA (p-<0.001).

**Table-II:** Frequency of HLA B27+ve and HLA B27-ve alleles in JIA patient and control groups and their association with JIA.

Variable		Patients (n=71) n(%)^a^	Control (n=34) n(%)	OR^b^ (95% CI)^c^	^d^P-Value
HLA -B27	+ve	27(38.0%)	30(88.2%)	0.082 (.026 - 0.258)	<0.001*
	-ve	44(62.0%)	4(11.8%)		

a number and percentage (frequency),

b odds ratio,

c confidence interval,

d p- value was determined by Pearson’s chi-square test,

* p≤ 0.05 is considered statistically significant.

Immune markers between HLA B27 positive and HLA B27 negative subjects of study groups are presented in Table-III. Regarding absolute count of blood B cells, no statistically significant difference (*p*=0.13) was found in children with HLA B27 positive and negative allele of patient group, whereas HLA B27 positive control population with median (IQR) of 1.10 (0.70-1.50) and HLA B27 negative with median (IQR) of 0.75 (0.55-0.87) has statistically significant difference (*p*=0.03). For blood B cells percentage of HLA B27 positive and negative subjects in JIA patient group has no significant difference. On the other hand, in control group statistically significant difference (*p*=0.01) was found between HLA B27 positive and HLA B27 negative subjects with median (IQR) of 26.55 (22.70-33.53) and 18.45 (14.80-21.05) respectively. For IL-1, IL-6 and TNF-α in the patient and control groups, subjects with HLA B27 positive allele and HLA B27 negative allele have no significant difference.

**Table-III:** Comparison of immune markers between HLA B27 +ve and HLA B27 -ve groups of patient and control populations.

Variables	Patient Group N=71		^a^p- Value	Control Group N=34		^a^p-Value

	HLA B27+ve	HLA B27-ve		HLA B27+ve	HLA B27-ve	
Absolute Count of Blood B-cells Median (IQR)	0.50 (0.37-0.80)	0.60 (0.32-0.87)	0.13	1.10 (0.70-1.50)	0.75 (0.55-0.87)	0.03
% of B Cells Median (IQR)^b^	22.50 (14.40-26.70)	19.70 (13.85-27.15)	0.60	26.55 (22.70-33.53)	18.45 (14.80-21.05)	0.01
Serum IL-1 (pg/ml) Median(IQR)	15.60 (13.10 - 30.60)	15.15 (12.55- 18.65)	0.68	13.80 (12.80 - 17.40)	13.45 (13.17 - 13.87)	0.45
Serum IL-6 (pg/ml) Median(IQR)	65.00 (49.90 - 92.70)	59.55 (43.72 - 85.85)	0.57	46.00 (36.10 - 60.40)	36.25 (30.27 - 43.35)	0.18
TNF α (pg/ml) Median(IQR)	57.20 (46.90 - 115.30)	50.45 (31.60 - 125.95)	0.47	43.00 (31.30 - 81.40)	51.10 (30.07 - 626.97)	0.77

a p-value = determined by Mann-Whitney U test,

b Inter-quartile range, *p≤ 0.05 is considered statistically significant.

## DISCUSSION

The current study found a statistically significant difference in HLA B27 frequency and a percentage between the control and patient groups with more HLA B27 alleles in the control group. These results showed that HLA B27 is a protective factor and it is linked with a low risk of JIA development in the native population. HLA B27 prevalence varies greatly across the globe, from nil in Australian aborigines to 50% in Haida Indians, with 6-8% of the Indian subcontinent having it.^3^,^9^ Studies of HLA B27 have been conducted in Pakistan showing an overall prevalence of 1.3%, while Pathan (9.4%) and Kalash (4.2%) had higher frequencies than rest of the population.^10^,^11^ Similarly, in a study on the Pakistani population by Lodhi et al., the HLA B2707 allele was more frequent in the healthy population in comparison to AS patients, whereas the B2706 allele was present only in the healthy group.^12^ All these findings augment the results of the current study showing presence of HLA B27 more prevalent in healthy populations than in JIA patients, possibly due to the geographical distribution of the gene.

In a study by Stanevicha et al., HLA B27 antigen was linked to JIA-ERA, oligoarthritis and polyarthritis.^13^ Similarly, according to another study HLA B27 antigen is a significant risk factor for the development of enthesitis-related arthritis, psoriatic, and oligoarthritis, with little impact on the initiation of the disease.^14^ The findings of the current study are contradictory to these studies due to the geographical and ethnic variations in the prevalence of HLA B27 allele. Another reason for the contradictory results may be that the prevalence of HLA B27 in a population is not always associated with the development of arthropathies. For example, Japanese population with low prevalence of HLA B27 have a strong SpA association, while UK populations with 8% of the general population carry HLA B27 but only 1% develop the diseases.^9^

Unlike, in a study by Varnavidou-Nicolaidou A et al., in the Greek Cypriot population a possible protective role of the B 2707 allele against B27-related diseases was found. Moreover, HLA-B 2707 allele was also detected in the healthy Greek population as well.^15^ HLA B27 protects against JIA in this cohort, but more genetic studies in various ethnic groups will contribute to the existing data about the negative or positive disease association with HLA B27 and its suballeles.

In this study on comparison of peripheral blood B cell counts and percentages the difference was significant between the HLA B27 positive and HLA B27 negative control subjects, with B cells being low in the HLA B27 positive control group. However, peripheral blood B cell counts and percentages were not different between the HLA B27 positive and HLA B27 negative patient groups. According to a study by Christiansen et al, no significant differences between the groups (B27 positive individuals with AS, B27 negative relatives without AS, B27 negative relatives without AS) was found in either the percentage or actual number of B lymphocytes.^16^ B cells are involved in the pathogenesis of JIA, producing autoantibodies, cytokines, antigen presentation, and activating T cells.^17^ A number of genes affected in JIA; can be expressed by B cells, thereby potentially influencing their functional role.^18^ Future research should focus on understanding the role of B cells in JIA disease diagnosis, prognosis, and treatment responses.

In this study serum cytokines i.e IL-1, IL-6 and TNF-α showed more concentrations in HLA B27 positive patient group as compared to HLA B27 negative patient group but the difference was not significant. However, IL-1, IL-6 have more serum concentration in HLA B27 positive control group but for TNF-α it is more in HLA B27 negative control group in comparison of HLA B27 positive group with no significant difference. HLA B27 plays a crucial role in immune pathogenesis, with the HLA B27 misfolding hypothesis suggesting that aberrantly folded HLA B27 accumulates in the endoplasmic reticulum, activating the unfolded protein response and triggering signaling cascades. This leads to the production of ER resident chaperones (BiP), potentially causing inflammation in macrophages. Another pathway that can activate ER stress causing ER-overload response (EOR) is triggered by excessive membrane protein trafficking, triggering the transcription of NF-κB, which can increase the production of proinflammatory cytokines like TNF-α, IL-1, and IL-6.^8^,^19^,^20^

Our results are in consistent with a study in which the expression of IL-23, IL-12 and TNF-α by peripheral blood-derived macrophages were higher in HLA B27 positive SpA in comparison with healthy donors.^8^ Similarly, according to another study Lipopolysaccharide-exposed cells of HLA B27 positive and negative recovered Yersinia arthritis patients, and HLA B27 positive controls, generated significantly more TNF-α and IL-1 than did HLA B27 negative control cells.^21^ In contrast, according to a study by Keller et al. twenty five HLA B27 positive patients with AS, as well as 18 healthy HLA B27 positive controls and 22 healthy HLA B27 negative controls were studied and showed a reduction in Th1 cytokines (IFN-γ, TNF-α) in HLA B27 positive patients, including healthy controls.^22^

### Limitations:

In the present study circulating B-lymphocytes were quantitatively assessed, but functional testing by antibody production was not done. The analysis of cytokine in plasma of JIA patients should be interpreted carefully because plasma cytokines are always a reflection of the complex immunological mechanisms of the disease. Parallel investigation of the immunological markers in synovial fluid is also missing as performing an articular puncture was not ethically acceptable. Nowadays sensitive kits are available for HLA profiling but due to financial constrain conventional approach was used to detect HLA B27 allele.

## CONCLUSION

HLA B27 is more in the healthy population as compared to disease group indicated HLA B27 as a protective allele to develop JIA in native population. Moreover, B cells and serum cytokines showed no significant difference in JIA patients with HLA B27 positive and HLA B27 negative allele. However, peripheral blood B cell counts and percentages showed significant difference between the HLA B27 positive and negative control subjects, with B cells being low in the HLA B27 positive control group. Further research is needed for better understanding of the immunological mechanisms involved in associations between HLA B27 allele and JIA disease, leading to the development of good practice guidelines.
